# High throughput profiling of the B cell repertoire identifies systematic changes in the repertoire of individuals with Crohn’s disease

**DOI:** 10.3389/fimmu.2026.1725813

**Published:** 2026-02-06

**Authors:** Aya K. H. Mahdy, Zahra Taheri, Marte Lie Høivik, Andre Franke, Hesham ElAbd

**Affiliations:** 1 Institute of Clinical Molecular Biology, Kiel University and University Hospital Schleswig-Holstein, Kiel, Germany; 2 Department of Biology and Biotechnology, University of Pavia, Pavia, Italy; 3 Department of Gastroenterology, Oslo University Hospital, Oslo, Norway; 4 Institute of Clinical Medicine, Faculty of Medicine, University of Oslo, Oslo, Norway; 5 Institute for Digestive Research, Lithuanian University of Health Sciences, Kaunas, Lithuania

**Keywords:** B cell, B cell repertoire sequencing, IBD, Ig isotype, somatic hypermutation, treatment-naïve

## Abstract

The B cell repertoire contains the recombined sequences that encode the entire antibody repertoire of an individual. The repertoire is made from three antigenic binding chains, namely the immunoglobulin heavy chain (IGH) and two immunoglobulin light chains, κ (IGK) and λ (IGL). Compared to the T cell repertoire, the B cell repertoire is understudied in inflammatory bowel diseases (IBD) even though different antibodies such as ASCA (Anti-Saccharomyces cerevisiae) and ANCA (Anti-Neutrophil Cytoplasmic Antibodies) have been shown to be elevated in individuals with IBD. To address this limitation, we profiled the B cell repertoire of peripheral blood from 27 treatment-naive individuals with CD and 21 age-matched symptomatic controls using bulk B cell receptor sequencing. The repertoire of individuals with CD showed a reduction in diversity and an increase in clonality. Furthermore, we observed a significant reduction in the expansion of IgM and IgD and an expansion of IgA2, and IgG2 clonotypes in individuals with CD relative to controls, suggesting an antigen-driven expansion. This was also supported by higher levels of somatic hypermutations, particularly in the complementary determining region 2 (CDR2) of immunoglobulin heavy chain, in individuals with CD relative to the control group. Thus, despite the small sample size, we identified multiple alterations in the B cell repertoire of individuals with CD, highlighting the potential of the B cell repertoire in identifying antigenic exposures implicated in the disease, demanding now larger international studies, ideally including also treatment-naive and pre-clinical cases.

## Introduction

Inflammatory bowel disease (IBD) is an incurable immune-mediated inflammatory disease that predominantly affects the gastrointestinal tract (GIT). It is observed clinically in two main forms: Crohn’s disease (CD), which is characterized by patchy transmural inflammation of different sections of the GIT, predominantly the ileum and the colon, and ulcerative colitis (UC), which is restricted to the colon. The exact cause(s) of IBD remain unknown, however, different genetic variants have been associated with the disease, such as *ATG16L* (1, 2), and *NOD2* (3). Additionally, several human leukocyte antigen (HLA) alleles have been implicated in IBD, such as HLA-DRB1*03:01 (4), or in a specific subset of individuals with ileal CD, such as HLA-DRB1*07:01 (4) and HLA-DRB1*15:01 in UC (5). Besides genetic predispositions, other risk factors have been identified, *e.g.*, smoking, microbial dysbiosis (6), antibiotic intake (7), and previous episodes of infectious mononucleosis (8), which is mainly caused by an Epstein-Barr virus (EBV) infection.

From an immunological perspective, different alterations and dysregulated processes have been identified in individuals with IBD, including dysregulated responses toward the gut microbiome (9) and mycobiome (10). We previously observed a significant expansion of a subset of type II natural killer T cells, termed Crohn’s-associated invariant T (CAIT) cells, in individuals with CD (11, 12). Elevated antibody responses against bacterial flagellins (13) and several human herpesviruses, predominantly EBV, have been reported by others (14). Recently, we also performed a large-scale analysis of the T cell repertoire of individuals with IBD in comparison to matching controls, identifying thousands of clonotypes that were significantly expanded in individuals with IBD (15, 16). These disease-associated clonotypes represent an immunological fingerprint for common antigenic exposures implicated in the disease. Nonetheless, these antigens remain to be identified as T cell repertoire sequencing enables the identification of clonotypes, *i.e.*, V(D)J recombination sequences forming the T cell receptor, and not the exact antigens presented by HLA proteins and recognized by these T cells (17).

The dependency of T cells on HLA proteins, which are highly polymorphic (18), renders analysing the T cell repertoire a challenging task, specifically in case-control studies where thousands of samples are needed to pinpoint specific T cells involved in the disease across different HLA contexts (15, 16, 19). Thus, identifying B cells recognizing the same antigens across multiple individuals with CD might require a smaller sample size as B cell receptors (BCRs) recognise their antigenic targets directly, independent of any presenting molecules, *e.g.*, HLA proteins. Relative to the T cell repertoire, which has been studied by others (20–23) and us (11, 15, 19), the B cell repertoire of individuals with IBD remains under-investigated.

The B cell repertoire is a composite of all immunoglobulins heavy and light chains present in a sample, such as peripheral blood. These chains are generated from somatic recombination processes termed V(D)J recombination that generate chains with enormous sequence diversity. This diversity is also augmented by a unique process that happens in B cells, termed somatic hypermutation (SHM), where random single-nucleotide polymorphisms (SNPs) are introduced into the formed immunoglobulin chains to increase their affinity toward a particular antigen. The Ig heavy chain (IGH) has five main isotypes, namely, μ, δ, ϵ, α, and γ, which form the IgM, IgD, IgE, IgA, and IgG, respectively. Furthermore, the α and γ isotypes have different subclasses, namely, α1 and α2 that encode IgA1 and IgA2, and γ1, γ2, γ3, and γ4, which encode the IgG1, IgG2, IgG3, and IgG4 subtypes. There are two light chains, κ (IGK) and λ (IGL), but they only have one constant region and do not undergo class-switching.

Besides the seminal study by Bashford-Rogers et al. (24), which compared the BCR repertoire of six different autoimmune diseases, not many studies have investigated the immune repertoire in individuals with CD or UC. Scheid et al. (25) profiled the B cell repertoire of colonic tissues from individuals with UC and showed a bias from IgA1 and IgA2 isotype usage toward IgG2 usage in inflamed tissues. Similarly, Chen and colleagues (26) profiled the B cell repertoire of multiple tissues in individuals with IBD and showed dysregulated B cell responses in individuals with CD. Lastly, Kotagiri et al. (27) also profiled the repertoire of individuals with IBD and healthy controls and identified BCRs shared among individuals with CD.

Here, we aimed at investigating the B cell receptor of 27 treatment-naive individuals with CD and 21 matching symptomatic controls from the Norwegian inception cohort IBSEN-III (28). Symptomatic controls are individuals with symptoms of inflammatory bowel disease, but their endoscopic and radiological results excluded IBD as a potential cause for their symptoms.

## Results

### The B cell repertoire of treatment-naive individuals with CD exhibits systematic differences in clonality and diversity

**Figure 1:**
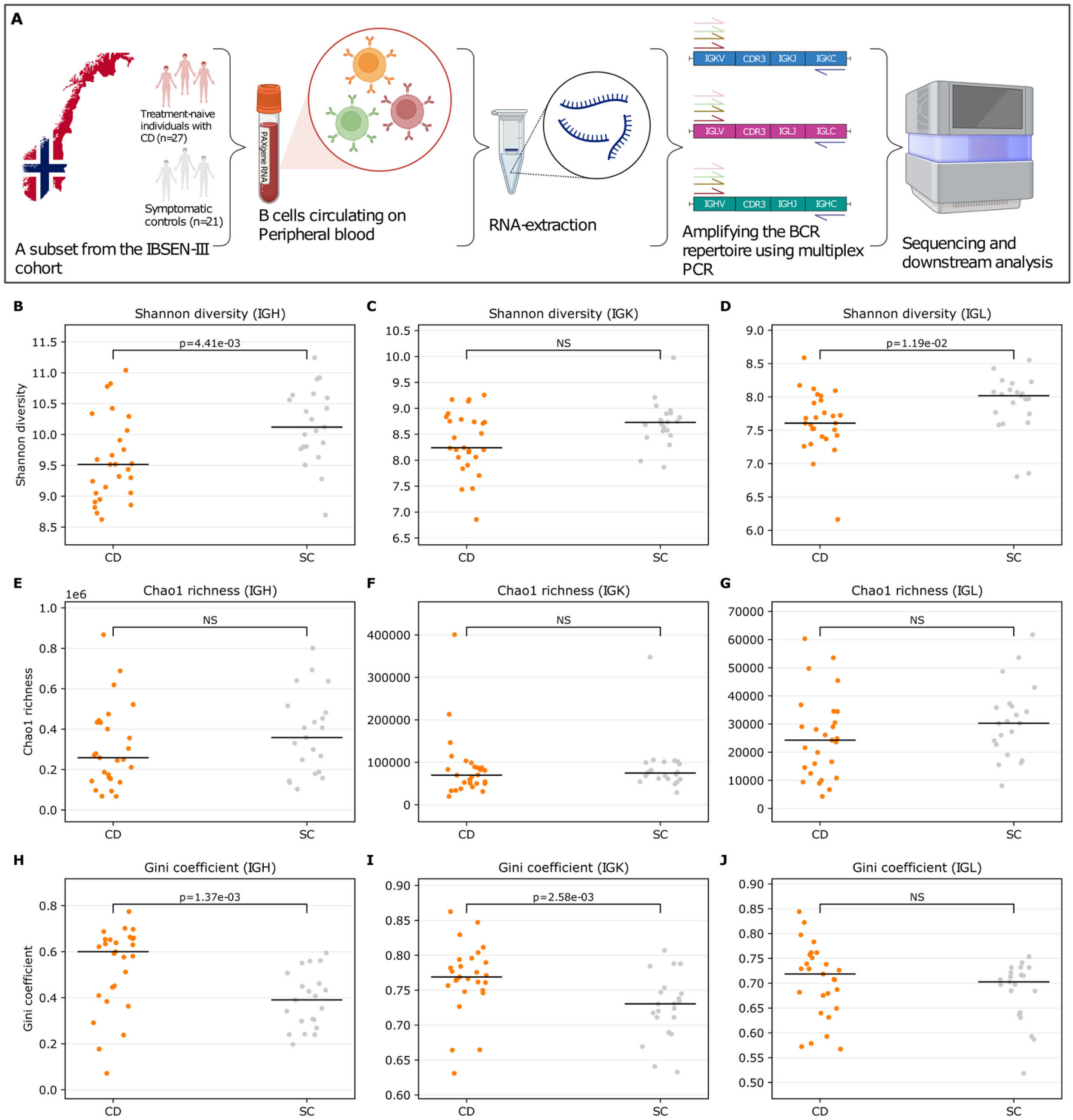
Repertoire diversity and inequality across BCR loci (productive clonotypes). **(A)** Schematic overview of the IBSEN-III peripheral blood BCR-seq workflow. Treatment-naive individuals (Crohn’s disease (CD) and symptomatic controls (SC)) were sampled; peripheral blood was collected into PAXgene tubes, RNA was extracted, and the BCR repertoire was amplified using multiplex PCR targeting IGH, IGK, and IGL rearrangements prior to sequencing and downstream analysis. Per-sample diversity metrics were calculated from productive clonotypes for the heavy chain (IGH) and light chains (IGK, IGL) and compared between Crohn’s disease (CD; orange) and control subjects (SC; grey). **(B–D)** Shannon diversity index (UMI-weighted clonotype abundance) for IGH, IGK, and IGL, respectively. **(E–G)** Chao1 richness estimator for IGH, IGK, and IGL, respectively. **(H–J)** Gini coefficient of the clonotype abundance distribution (higher values indicate greater clonal inequality/expansion) for IGH, IGK, and IGL, respectively. Each dot represents one sample; horizontal black lines indicate the median. P-values are from two-sided Mann-Whitney U tests (NS, not significant). Panel **(A)** was Created in BioRender. ElAbd, H. (2026) https://BioRender.com/cj89mj7.

We profiled the peripheral blood B cell receptor repertoire of 27 treatment-naive individuals with Crohn’s disease (CD) and 21 symptomatic controls from the Norwegian inception cohort IBSEN-III (**Table 1**, **Figure 1A**). RNA was extracted from PAXgene Blood RNA tubes, and IGH, IGK and IGL transcripts were amplified by multiplex PCR and sequenced in pooled libraries. Demultiplexed reads were processed with MiXCR (29) to assemble productive clonotypes derived from the three loci (Methods). We first assessed per-sample repertoire diversity and inequality across IGH, IGK, and IGL. Shannon diversity was reduced in CD relative to SC for IGH and IGL, while IGK showed no significant difference (**Figures 1B–D**), indicating a selective loss of repertoire evenness rather than a uniform effect across loci. Chao1 richness did not differ significantly between groups for any locus (**Figures 1E–G**), arguing against a broad reduction in the number of detectable clonotypes in CD. In contrast, the Gini coefficient, a direct measure of inequality in clonotype abundance, was higher in CD for IGH and IGK but not for IGL (**Figures 1H–J**), consistent with increased dominance of expanded clonotypes in CD. Together, these results support a model in which CD repertoires are primarily distinguished by increased clonal skewing/oligoclonality, with comparatively preserved estimated richness.

**Table 1:** Clinical and demographic characteristics of the study cohort.

	Crohn*’*s disease	Symptomatic controls
Number of individuals	27	21
Age (mean ± SD)	(27 *±* 6.2)	(27.9 *±* 6.57)
Percentage of females	44.4% (12 out of 27)	71.4% (15 out of 21)
Disease location (Number of individuals)	L1 (Ileum): (n=6) L2 (Colon): (n=7) L3 (Ileo-colon): (n=14) L4 (Upper GI tract): (n=2)	NA
Disease modifiers (Number of individuals)	B1 (non-stricturing, non-penetrating): (n=16) B2 (Stricturing): (n=7) B3 (Penetrating): (n=2)	NA

Summary of participant metadata for individuals with Crohn’s disease (n = 27) and symptomatic controls (n = 21), including age (mean ± SD) and sex distribution. Symptomatic controls were individuals presenting with symptoms suggestive of inflammatory bowel disease, but with endoscopic and radiological evaluations excluding IBD as the cause of their symptoms. For Crohn’s disease patients, disease location (L1-L4) and disease behavior/modifiers (B1-B3) are reported according to the Montreal classification; participants could be assigned to L4 in addition to another location category. Disease location and modifiers are not applicable (NA) for symptomatic controls.

### BCR repertoires are largely private and reflect individual antigen exposure

Motivated by the increased clonal dominance observed in Crohn’s disease (CD), we next asked whether repertoires share convergent clonotypes across individuals. To quantify between-sample overlap in a way that tolerates minor sequence variation, we compared repertoires using CDR3 amino-acid (CDR3-aa) similarity, defining two clonotypes as overlapping if their CDR3-aa sequences had the same length and differed by at most one amino-acid substitution (Hamming distance ≤1). For each locus (IGH, IGK, IGL), we computed a pairwise “fuzzy” Jaccard overlap between all samples. Across all three chains, overlap between unrelated individuals was uniformly low, with Jaccard values near zero for nearly all sample pairs, indicating highly private repertoires and limited convergent CDR3-aa sharing (**Supplementary Figures S1–S3**). Notably, we did not observe any consistent block structure or clustering by disease status, supporting the conclusion that any shared CDR3-aa convergence is rare and not a dominant feature distinguishing CD from symptomatic controls (SC) in peripheral blood. These results recapitulate what has been observed with functional antibody repertoire profiling using phage-immunoprecipitation sequencing (PhIP-Seq) (30). This approach aims at identifying the antigens bound by the collection of antibodies present in a sample without identifying the sequence of the BCR encoding for these antibodies. Using PhIP-Seq, we (19, 31, 32) and others (33–35) have shown that most antigenic exposures are private, *i.e.*, not shared among individuals, corroborating our observations about the private nature of humoral immunity.

### CD does not alter the VDJ recombination landscape of the blood B cell receptor repertoire

Next, we aimed at quantifying the impact of CD on the abundance of different V and J gene combinations. Within the IGH repertoire, some VJ recombination pairs dominated the repertoire, for example, *IGHV3-23*/*IGHJ4* derived clonotypes (**Supplementary Figure S4**). The dominance of this VJ gene combination is expected, where across multiple studies, *IGHJ4* have been shown to be utilised by >40% of clonotypes (36), similarly, the IGHV3–23 is one of the most frequently utilised *IGHV* genes within the pool of naive and memory B cells (37). Hence, our finding corroborates previous studies showing the dominant role of the *IGHV3-23*/*IGHJ4* gene combination on both the naive and memory B cell compartments. Similar findings were detected at the IGK repertoire, where *IGKV3–20* paired with *IGKJ1* dominated the repertoire (**Supplementary Figure S5**), corroborating previous observations (38–40). Within the IGL repertoire, the *IGLV2–14* paired with *IGLJ2* formed a high percentage of the IGL repertoire (**Supplementary Figure S6**) in individuals with and without CD, confirming previous reports about the dominant role of these genes in shaping the IGL repertoire (38, 41). Across the dataset, we identified 75 exact overlap records involving 44 samples. All overlaps were restricted to the IGL locus (IGL = 75; IGH = 0; IGK = 0) and mapped to a single PLAbDab entry (PDBmAb-24JAN2024-063013 (42);), with each positive sample contributing one or two matched clonotypes. Thus, within the “full FR1–FR4 exact overlap” definition, PLAbDab matches were detectable but rare and confined to light-chain lambda sequences in this cohort.

These findings indicate that CD induces a change in repertoire diversity and clonality, but it is not large enough to change the landscape of V(D)J recombination, at least not at a magnitude detected with our study’s statistical power. To extend this analysis to other less frequent VJ combinations, we compared the expansion of each unique VJ gene combination between individuals with and without CD (Methods). Nonetheless, after correcting for multiple testing, we could not detect any significant VJ gene combination that was significantly expanded in CD relative to SC across any of the three loci (**Supplementary Figure S7**).

### Individuals with CD have a significant reduction in the expansion of IgM and IgD clonotypes and an expansion of IgA2 and IgG2 clonotypes

**Figure 2:**
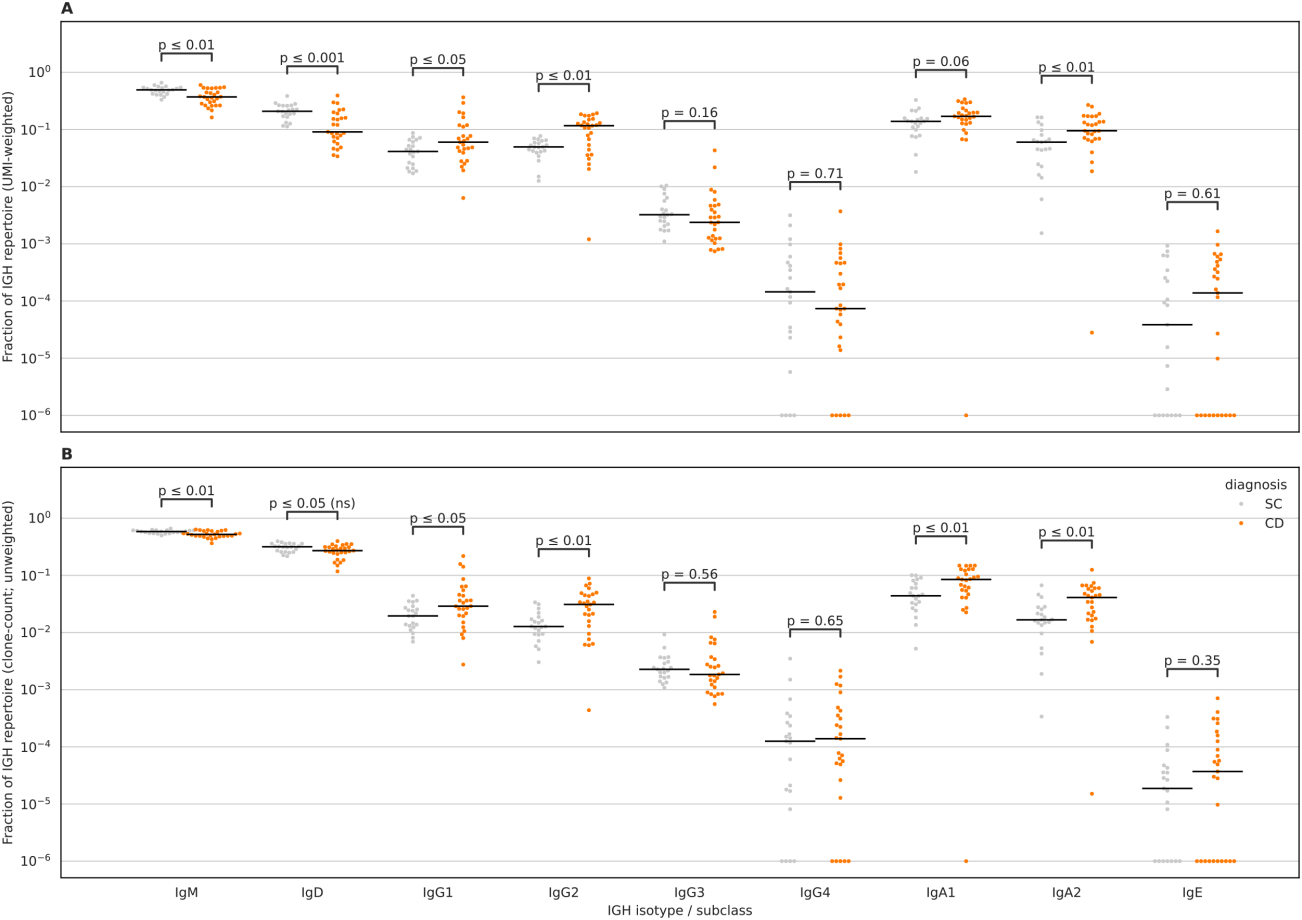
Differences in IGH isotype usage between Crohn’s disease (CD) and symptomatic controls (SC) using MiXCR-calculated unique-molecule fractions. **(A)** UMI-weighted isotype fractions per sample, computed by summing MiXCR calculated UMI fractions across clonotypes assigned to each IGH isotype/subclass (IgM, IgD, IgG1-4, IgA1–2 and IgE). **(B)** Clonotype-count (unweighted) isotype fractions per sample, computed as the fraction of unique clonotypes assigned to each isotype/subclass. Each dot represents one sample; horizontal black lines indicate group medians. CD samples are shown in orange and SC samples in grey. Statistical comparisons were performed per isotype using two-sided Mann-Whitney U tests with Benjamini-Hochberg (BH/FDR) correction across isotypes within each panel; adjusted p-values are shown above each isotype. Because axes are log-scaled, samples with missing or zero values were displayed at a small constant (1×10^-6^) for plotting only; raw values (including zeros) were retained for statistical testing.

**Figure 3:**
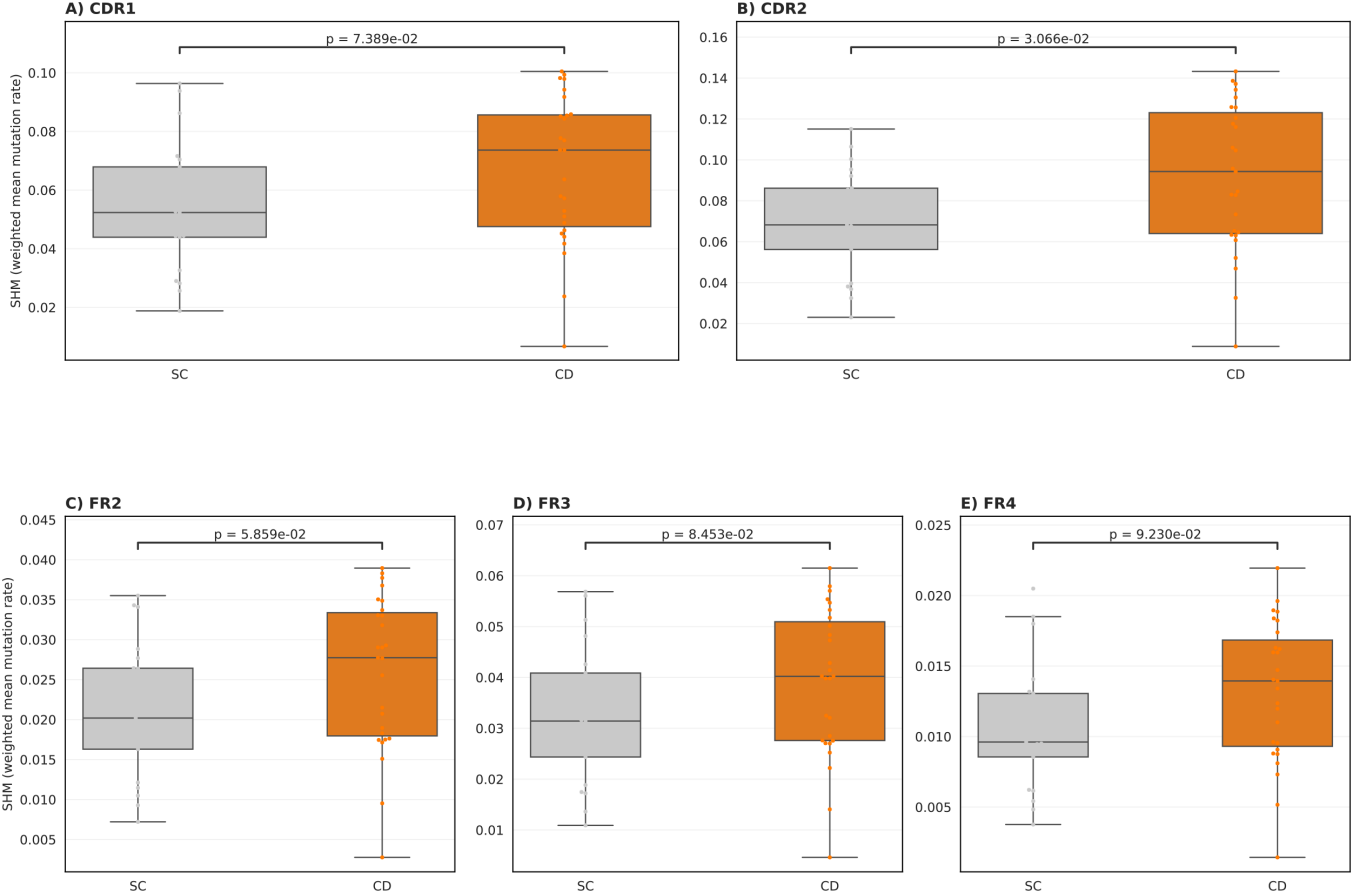
Region-resolved somatic hypermutation (SHM) in IGH differs between CD and SC. Per-sample, region-specific SHM rates were quantified from MiXCR allele-reassigned IGH clonotypes by comparing observed nucleotide sequences to their inferred germline counterparts for CDR1 **(A)**, CDR2 **(B)**, FR2 **(C)**, FR3 **(D)**, and FR4 **(E)**. For each sample and region, we computed a UMI-weighted mean SHM rate (mismatches/compared bases), where compared bases include only positions with unambiguous A/C/G/T in both observed and germline sequences. Each dot represents one sample (CD vs SC), with boxplots summarizing the distribution across samples (median and interquartile range). Statistical differences between CD and SC were assessed using a two-sided Mann-Whitney U test per region and corrected for multiple testing across regions using Benjamini-Hochberg (BH).

Subsequently, we aimed at investigating isotype chain usage by individuals with and without CD (Methods). We observed significantly lower levels of IgM and IgD in individuals with CD relative to SC (**Figure 2A**), similarly, we observed a significant increase in the levels of IgA2 and IgG2 in individuals with CD relative to SC (**Figure 2A**). This effect mostly persisted after removing the effect of expansion, *i.e.*, counting each clonotype as a singleton (**Figure 2B**). Thus, it shows that in CD there is a decrease in the number and expansion levels of IgM and an increase in the number and expansion levels of IgA2 and IgG2 clonotypes. To assess whether disease status was associated with altered affinity maturation, we quantified region-resolved IGH somatic hypermutation (SHM) by comparing observed clonotype sequences to their inferred germline counterparts after MiXCR allele reassignment. Across samples, SHM levels varied by region, with the largest values observed in the CDRs, consistent with antigen-driven selection. When comparing CD and SC, CD samples showed higher SHM levels overall, with the most pronounced separation observed in CDR2, which remained significant after FDR correction (**Figure 3B**). Differences in CDR1 and FR2 followed the same direction but did not reach significance after multiple testing correction (**Figures 3A, C**), while FR3 and FR4 exhibited broadly overlapping distributions between groups (**Figures 3D, E**). Together, these data indicate that disease status is associated with a modest but regionally concentrated increase in IGH SHM, strongest in CDR2.

## Discussion

By analysing peripheral blood B-cell receptor (BCR) repertoires from individuals with CD compared with symptomatic controls SC, we observed a shift toward expanded class-switched clonotypes in CD, most prominently within IgG2 and IgA2. IgG2 responses are classically enriched against carbohydrate/polysaccharide antigens and, relative to other IgG subclasses, often exhibit reduced Fc-mediated effector functions, including weaker complement activation and Fcγ-receptor engagement (43). In parallel, IgA2 is commonly associated with mucosal immunity and has been linked to recognition of commensal bacteria, consistent with heightened antigenic exposure at the gut interface. Together, the selective enrichment of IgG2/IgA2 clonotypes in CD is compatible with increased stimulation by microbial antigens, potentially facilitated by intestinal barrier dysfunction reported in CD. This interpretation is further supported by our SHM analyses, which indicate greater evidence of antigen-driven maturation in CD (notably in CDR2), alongside a less even repertoire. Our observations align with prior repertoire-level analyses reporting disease-associated remodelling of B-cell architecture in immune-mediated conditions including CD (24, 44).

Despite evidence of isotype-specific expansion, we detected limited sharing of exact clonotypes in the peripheral blood of the study participants. This is consistent with the strong tissue compartmentalization of B-cell responses and with recent work identifying disease-associated shared B-cell clones in lymph nodes rather than blood (27). In addition, while earlier reports suggested an IgE-associated signal in CD (24), we did not reproduce a robust IgE expansion in our cohort. Differences in the cohort size, analytical sensitivity, and genetic/environmental background may contribute to these discrepancies. Future studies with larger multi-ethnic cohorts will be important to clarify the contribution of IgE and other low-frequency isotypes to the pathogenesis of CD. Finally, we observed comparatively reduced expansion of IgM/IgD clonotypes in CD relative to SC, together with increased class-switched IgA/IgG clonotypes and elevated SHM. Collectively, these signatures support a model of increased antigenic encounter and/or immune priming in CD compared with symptomatic controls.

## Methods

### Cohort description

The cohort contains treatment-naive individuals with CD or symptomatic controls, *i.e.*, individuals with the symptoms of IBD but without endoscopic or radiological findings supporting their diagnosis. The study participants were recruited as a part of the Inflammatory Bowel Disease in South-Eastern Norway III (IBSEN III) study (28). The IBSEN-III study was approved by the South-Eastern Regional Committee for Medical and Health Research Ethics (Ref 2015/946-3) and performed in accordance with the Declaration of Helsinki.

### Profiling the B cell receptor

Whole blood was collected into PAXgene Blood RNA tubes (Qiagen) and total RNA was extracted from stabilized blood, that is, no cell sorting was performed and total RNA was used for profiling the repertoire. After quality controls, 300ng of RNA were used to profile the IGH, IGL and IGK repertoire of the samples using the Human IG RNA Multiplex kits from MiLaboratories according to the manufacturer’s instructions. This multiplex design employs V-gene-specific forward primers located within framework region 1 (FR1) together with constant-region primers, producing amplicons that start downstream of the 5’ portion of FR1. Consequently, assembled clonotype sequences reliably cover the variable-domain segments spanning CDR1-CDR3 and framework regions FR2-FR4, while FR1 is not fully captured by the assay. After library preparation, Illumina’s unique dual indexing adaptors were used to tag each sample with a unique index, then samples were pooled together and then cleaned with magnetic beads (AMPure XP, Beckman Coulter). Next, samples were sequenced using a 2×250 bp paired-end using a NextSeq 1000 at the Illumina Solutions Center in Berlin.

### Clonotype identification from sequencing reads

After sequencing and sample demultiplexing, paired-end reads from each sample were processed with MiXCR v4.7.0 (29) using the manufacturer-recommended preset for the MiLaboratories Human BCR RNA IG UMI Multiplex protocol. MiXCR performed read pre-processing, alignment to reference V(D)J gene segments, UMI-aware assembly, and clonotype calling. For each sample, clonotype tables were generated for the immunoglobulin heavy (IGH) and light chains (IGK and IGL). Clonotype tables were then exported from MiXCR as tab-delimited files for downstream analyses, including per-clone abundance metrics (read count/fraction and UMI-derived molecule counts/fractions) and gene annotations (V and J gene calls and constant gene/isotype assignment for IGH). Unless stated otherwise, downstream analyses were restricted to productive clonotypes to focus on functional immunoglobulin rearrangements.

### Diversity and inequality calculations

Diversity metrics were computed per sample and per locus from the distribution of clonotype abundances. For a given sample, let R denote the number of clonotypes (clonal groups) and let $n_{i}$ denote the abundance of clonotype i (UMI counts summed across sequences assigned to the clonotype). Abundances were converted to relative frequencies $p_{i}$ by normalizing to the total abundance $N=\sum_{i=1}^{R}n_{i}$, *i.e.*, $p_{i}=n_{i}/N$.

Shannon diversity was calculated as:


$$shannon\;diveristy=\;-\;\sum_{i=1}^{R}\;p_{i}*\log\left(p_{i}\right)\;$$

Chao1 richness was used as an estimator of the total clonotype richness accounting for under sampling:


$$S_{Chao1}=S_{obs}+\;\frac{f_{1}^{2}}{2\;f_{2}}$$

where $S_{obs}$ is the observed number of clonotypes in the sample, $f_{1}$ is the number of singleton-clonotypes (with $n_{i}=1$), and $f_{2}$ is the number of doubletons clonotypes (with $n_{i}=2$). When $f_{2}=0$, we used the standard bias-corrected form:


$$S_{Chao1}=S_{obs}+\;\frac{f_{1}\left(f_{1}-1\right)}{2\;(f_{2}+1)\;}$$

To quantify repertoire unevenness (oligoclonality), we calculated the Gini coefficient per sample from clonotype abundances. Let $n_{i}$ denote the abundance (UMI count) of a clonotype i and R the number of clonotypes in the sample. After sorting abundances in non-decreasing order ( $n_{\left(1\right)}\leq \cdots \leq n_{\left(R\right)}$) and defining $N=\sum_{i=1}^{R}n_{\left(i\right)}$, the Gini coefficient was computed as:


$$\text{Gini inequality }\left(\text{G}\right)\;=\;\;\frac{2\;\sum_{i=1}^{R}\;i\;n\left(i\right)}{R\;N}\;\;\;-\;\;\;\frac{R+1}{R}$$

where G∈[0,1], with G=0 indicating a perfectly even repertoire (all clonotypes have equal abundance) and higher values indicating increasing inequality consistent with oligoclonal expansion. Metrics were computed per sample and per locus using productive sequences only.

### Fuzzy CDR3 overlap analysis

To quantify inter-individual similarity while allowing for minor amino-acid variation, we computed a fuzzy repertoire overlap using CDR3 amino-acid (AA) sequences only for each locus (IGH, IGK, IGL). For each sample, CDR3 AA strings were extracted and collapsed to unique sequences. To define fuzzy groups, all unique CDR3 AA sequences across samples were clustered under a Hamming distance threshold of ≤1 substitution, restricted to sequences of identical length. Clustering was performed separately within each length group using a wildcard indexing approach. Each sample repertoire was then represented as the set of fuzzy cluster identifiers present in that sample. Pairwise sample overlap was quantified using the Jaccard index (intersection/union) computed on these cluster sets, producing a sample-by-sample overlap matrix per locus.

### Comparing V-J recombination frequencies between conditions

To compare V-J recombination usage between individuals with Crohn’s disease (CD) and symptomatic controls (SC), we first collapsed V and J calls to gene-level (alleles removed) and defined each V-J combination as V_gene-J_gene. For each sample, we quantified the frequency of each V-J pair as the summed UMI counts assigned to that V-J pair divided by the total UMI count in that sample for the corresponding locus. For each group (CD or SC), we computed the mean V-J frequency across samples:


$$\mu_{VJ}\;=\frac{1}{S}\;\sum_{i=1}^{s}\;f_{vj,i\;}+\;\epsilon \;\;\;\;\;\;\;\;\;\;\;\;\;$$

where $f_{VJ,i}$ is the per-sample frequency of the V-J pair in sample i, S is the number of samples in the group, and ϵ is a pseudo-count ( $\epsilon =1\times 10^{-8}$) to accommodate V-J pairs not observed in a given group. We then computed the log2 fold change between groups:


$${fold\;change\;}_{VJ}\;={\text{log}}_{2}\left(\frac{\mu_{VJ}\;CD}{\mu_{VJ}\;SC}\right)\;\;\;\;\;\;\;\;\;\;\;$$

For each V-J pair, the statistical significance of differences in per-sample frequencies between CD and SC was assessed using a two-sided Mann-Whitney U test, followed by Benjamini-Hochberg correction to control the false discovery rate.

### Similarity to characterized antibodies (PLAbDab)

BCR repertoires were processed with MiXCR (v4.7.0) (29), including the ‘findAlleles’ command, and clone tables were generated from the resulting files. For each sample and each locus (IGH, IGK, IGL), clonotypes were exported using ‘mixcr exportClones’ to retain clonotype identifiers and abundance metrics, namely, (‘cloneId’, ‘readCount’, ‘uniqueMoleculeCount’, and ‘uniqueMoleculeFraction’), together with imputed amino-acid sequences across the variable domain using ‘-allAAFeaturesImputed FR1Begin FR4End’. For every sample in a given locus, clonotypes were ranked by UMI abundance, and the top 1000 expanded clonotypes were retained. The imputed FR1-FR4 amino-acid sequence per clonotype was defined as the concatenation of the exported framework and CDR segments spanning FR1 through FR4 (“full FR1-FR4 overlap” string). To assess public/known antibody overlap, PLAbDab (45) paired and unpaired sequence exports were ingested, sequences were cleaned to standard amino-acid strings, and light-chain vs heavy-chain entries were assigned to the appropriate locus. Exact overlaps were then computed by strict string equality between each MiXCR clonotype’s full FR1-FR4 amino-acid sequence and the PLAbDab (45) variable-domain amino-acid sequence field. All matches were written to a consolidated overlap table including MiXCR abundance metrics and PLAbDab record metadata.

### IGH isotype/subclass utilization analysis

We quantified IGH constant-region usage per sample from MiXCR clonotype exports. Constant-region annotations (isotype) were standardized by (i) taking the first call when multiple were present, (ii) removing allele suffixes (e.g., *00, *01), and (iii) harmonizing IMGT-style labels to a fixed set and order: IgM, IgD, IgG1-4, IgA1-2 and IgE. Only clonotypes assigned to these categories were retained. For the UMI-weighted analysis, we used MiXCR ‘uniqueMoleculeFraction’ (per-clonotype fraction of unique molecules within a sample). For each sample and isotype, we summed uniqueMoleculeFraction across clonotypes, yielding a per-sample isotype fraction reflecting both isotype composition and clonal expansion. For the clone-count (unweighted) analysis, we counted unique clonotypes per isotype in each sample and divided by the total number of clonotypes in the sample, yielding an isotype fraction reflecting isotype diversity independent of expansion. Group comparisons (CD vs SC) were performed independently for each isotype using two-sided Mann-Whitney U tests, and p-values were adjusted using Benjamini-Hochberg FDR correction across isotypes within each panel. For visualization on log-scaled axes, missing/zero values were displayed at 1×10^–6^ for plotting only, while the raw values (including zeros) were used for statistical tests.

### Somatic hypermutation analysis

- MiXCR allele inference and clonotype export. IGH repertoires were processed with MiXCR and subjected to allele inference using ‘findAlleles**’**, generating allele-reassigned clonotype tables per sample. For downstream SHM quantification, clonotypes were exported with MiXCR to include: (i) clonotype identifiers and abundance, and (ii) nucleotide sequences for immunoglobulin subregions along with their corresponding inferred germline sequences.
- Per-sample SHM computation. SHM was computed from the exported clonotype tables using a custom Python workflow. For each sample and for each region (CDR1, CDR2, FR2, FR3, and FR4), the analysis script, compares the observed nucleotide sequence column (*e.g.*, nSeqCDR1) to the corresponding germline column (*e.g.*, nSeqCDR1OfGermline). Only positions where both sequences contain unambiguous A/C/G/T bases are considered “compared bases”; positions with ambiguous characters are excluded. For each clonotype, the number of mismatches and compared bases are calculated, and then aggregated to the sample level using abundance weights:
  - Weighted mismatches = sum (weight x mismatches).
  - Weighted compared bases = sum (weight x compared bases).
  - Weighted mean SHM rate = (weighted mismatches)/(weighted compared bases).

The script also reports the fraction of clonotypes with valid coverage for each region (found fraction) and the total weighted compared bases per region. The final output is a per-sample table containing region-specific weighted mean SHM values used for plotting and group comparisons. For each region, per-sample weighted mean SHM rates were compared between CD and SC using a two-sided Mann-Whitney U test. Multiple testing across regions was controlled using Benjamini-Hochberg correction. Boxplots and sample-level points were produced from these per-sample SHM summaries, and BH adjusted p-values were displayed in the figure.

## Data Availability

The datasets presented in this article are not readily available because of data privacy regulations in Norway and our institution. However, data are available upon request, if the aims of the planned analyses are covered by the written informed consent signed by the participants, pending an amendment to the ethical approvals and a material & data transfer agreement between the institutions. Requests to access the datasets should be directed to Marte Lie Høivik, m.l.hoivik@medisin.uio.no.
