# Supplementary material for: High throughput profiling of the B cell repertoire identifies systematic changes in the repertoire of individuals with Crohn’s disease
Source: Front Immunol. 2026 Feb 6;17:1725813. doi: 10.3389/fimmu.2026.1725813 (PMC12920447; doi:10.3389/fimmu.2026.1725813)
Supplement: Supplementary file 1 [file DataSheet1.pdf]

Supplementary figures

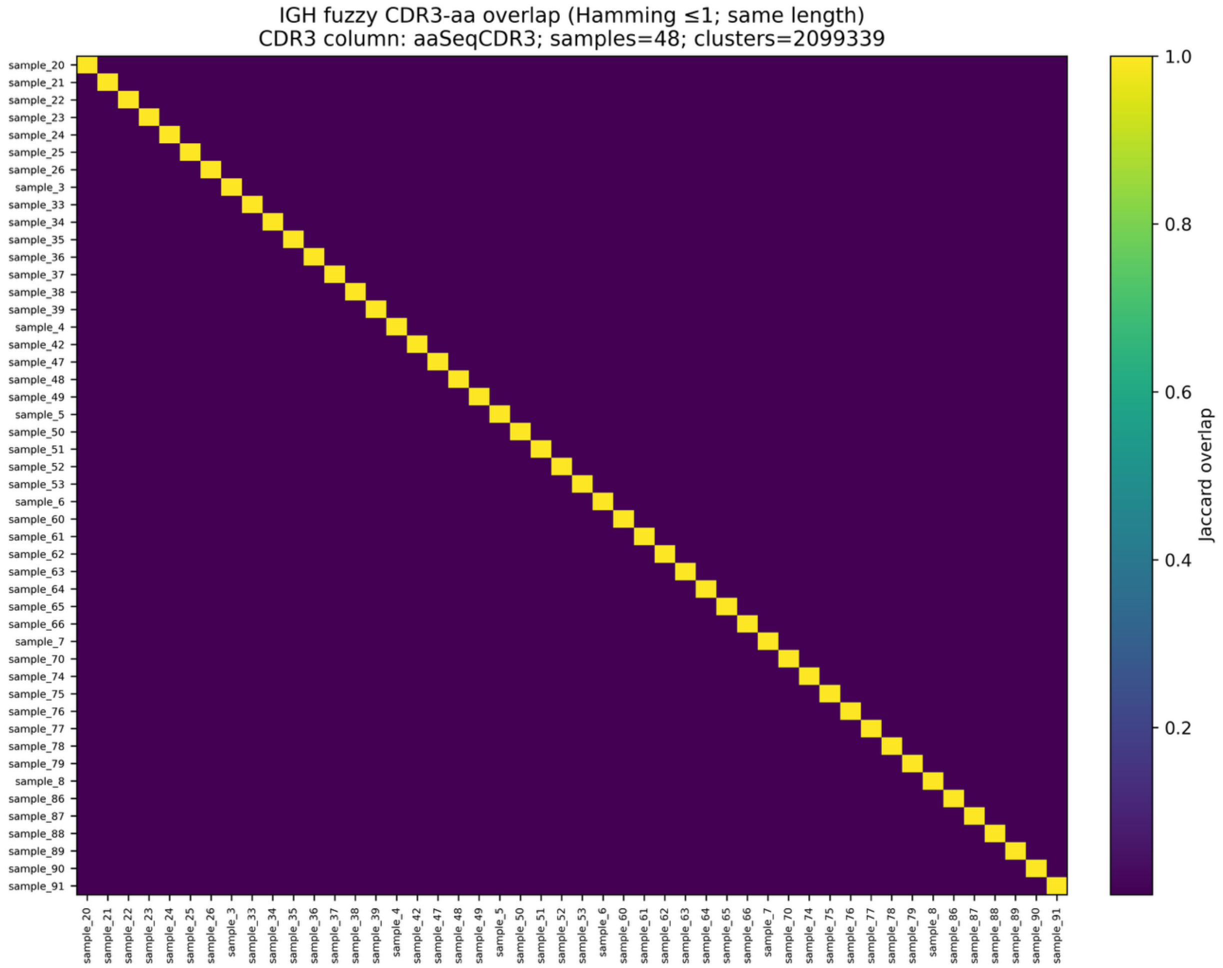

Figure S1: Limited overlap of IGH repertoires using fuzzy CDR3-aa similarity. Heatmap showing pairwise repertoire overlap between samples for the IGH locus, quantified by a fuzzy Jaccard index computed on CDR3 amino-acid sequences. Two CDR3-aa sequences were considered matching if they had the same length and differed by ≤1 amino-acid substitution (Hamming distance ≤1). Each cell reports the Jaccard overlap (intersection/union) between the two samples' fuzzy CDR3-aa sets; diagonal entries equal 1. Sample order is identical on both axes. Overall, off-diagonal values are close to zero, indicating minimal IGH CDR3-aa sharing between individuals.

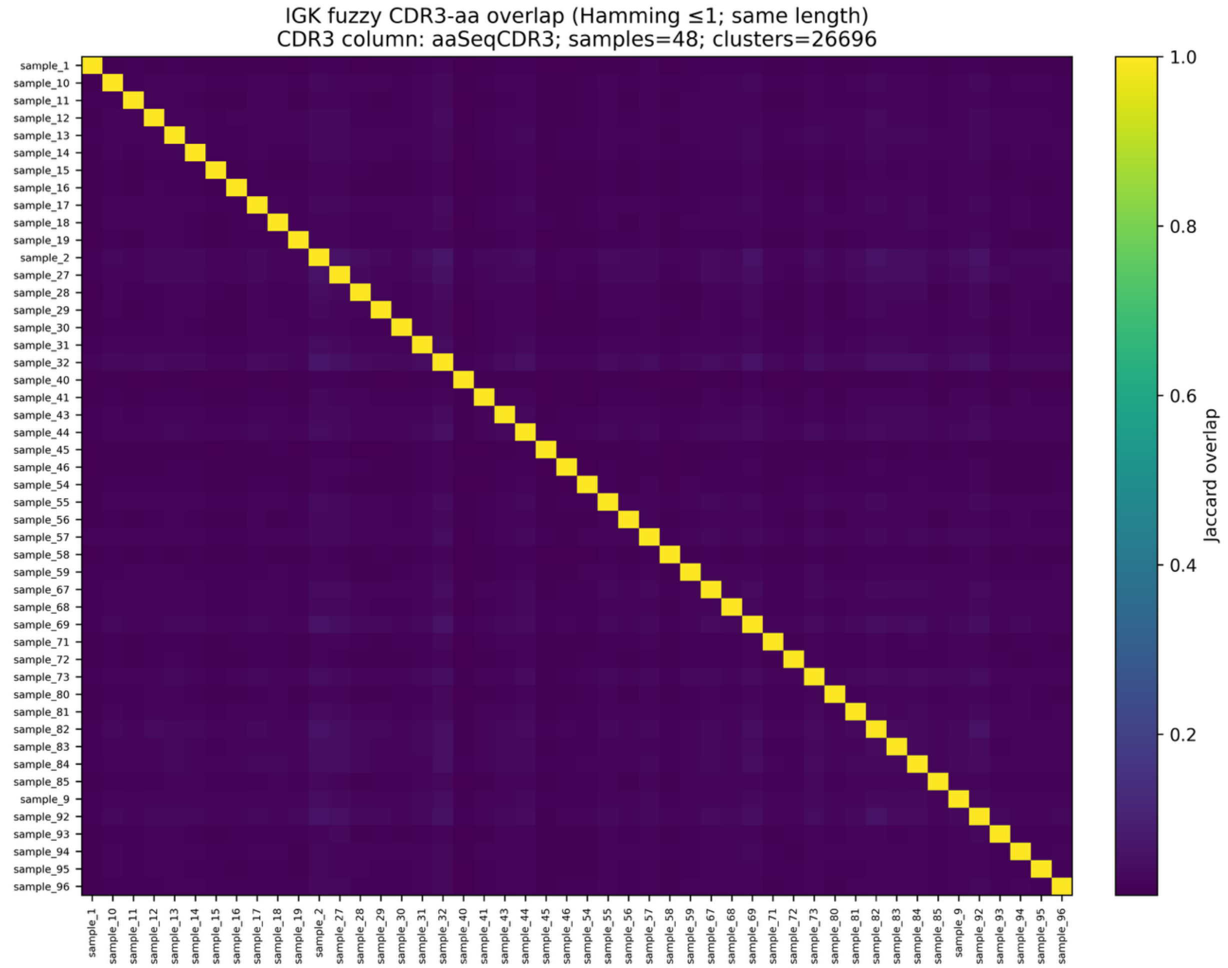

Figure S2: Limited overlap of IGK repertoires using fuzzy CDR3-aa similarity. Heatmap of pairwise **IGK** repertoire overlap across samples, computed as a fuzzy Jaccard index on CDR3-aa sequences with same-length and Hamming distance ≤1 matching. Diagonal values represent within-sample overlap (1.0), whereas off-diagonal values are uniformly low, demonstrating that IGK repertoires are largely private with limited convergent sharing of closely related CDR3-aa sequences.

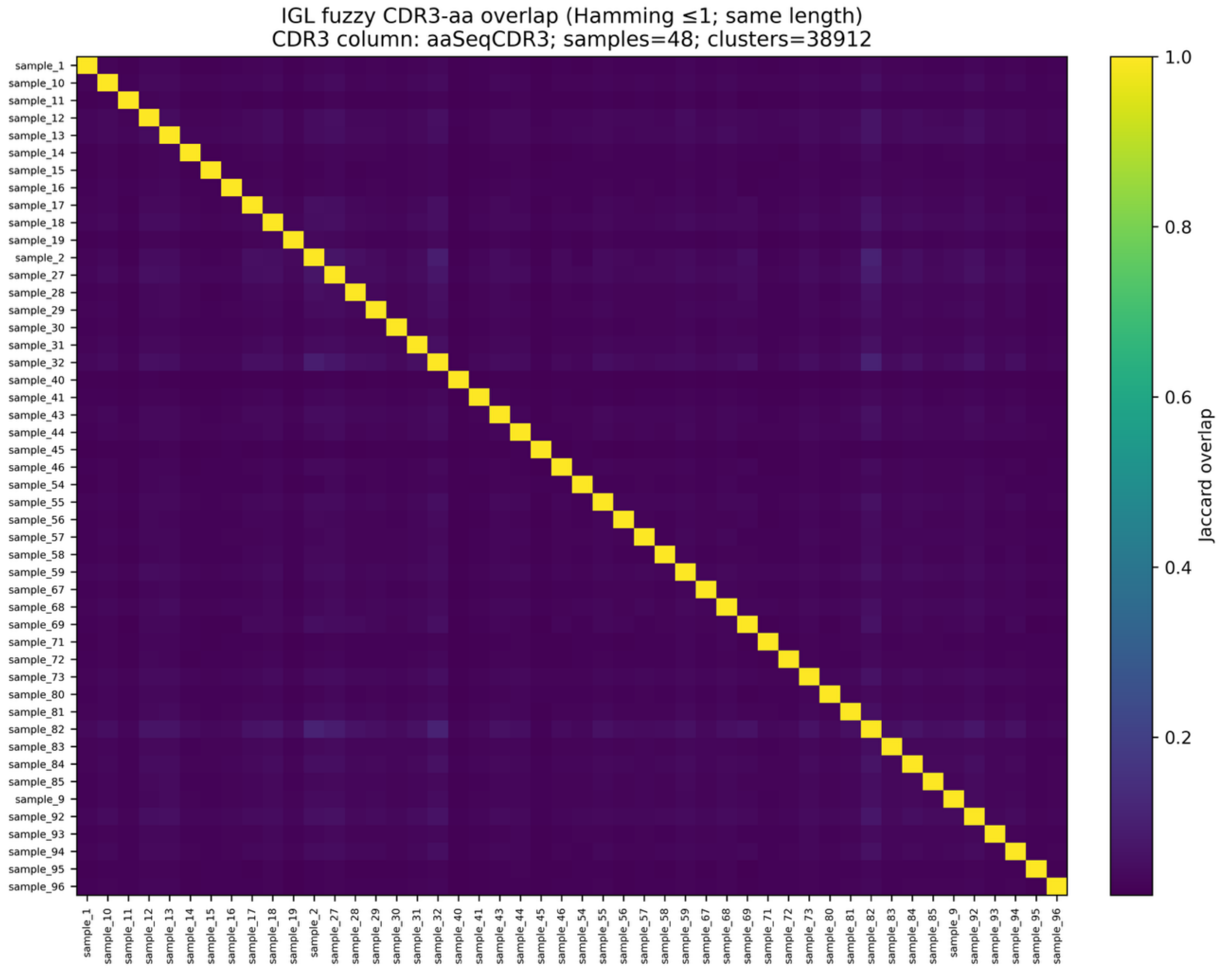

Figure S3: Limited overlap of IGL repertoires using fuzzy CDR3-aa similarity Heatmap of pairwise **IGL** repertoire overlap across samples using a fuzzy Jaccard index defined on CDR3-aa sequences. Overlap was assessed allowing ≤1 amino-acid substitution (Hamming distance ≤1) between same-length CDR3-aa sequences. Off-diagonal values are near zero across most sample pairs, indicating minimal sharing of IGL CDR3-aa motifs between individuals and no clear clustering by disease status.

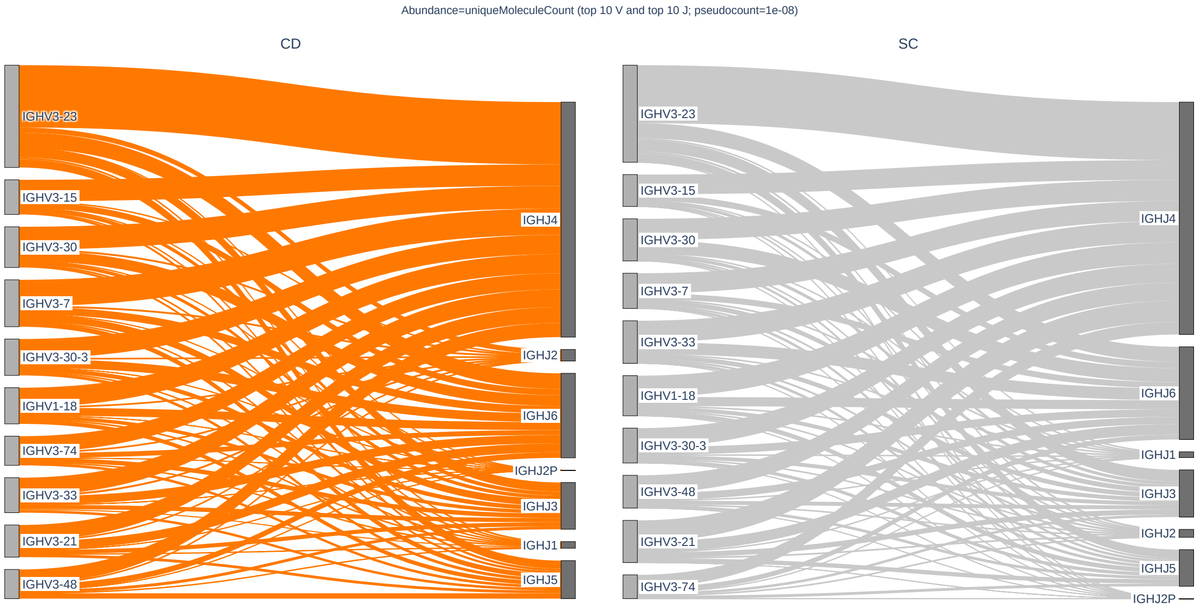

Figure S4*:* V-J gene usage in the IGH repertoire of Crohn's disease (CD) cases and symptomatic controls (SC). Sankey diagrams summarize IGH V-J recombination patterns aggregated across all productive IGH sequences in the cohort. For each diagnosis group (CD, left; SC, right), node labels represent the top 10 IGHV genes (left column of nodes) and the top 10 IGHJ genes (right column of nodes) ranked by total abundance across the entire cohort (CD+SC). Link widths are proportional to the summed abundance for each V-J pair within the indicated group (UMI counts), with a pseudo-count of 1×10^{-8}added to all V-J combinations to avoid zero values. Link colors denote diagnosis (CD, orange; SC, light grey).

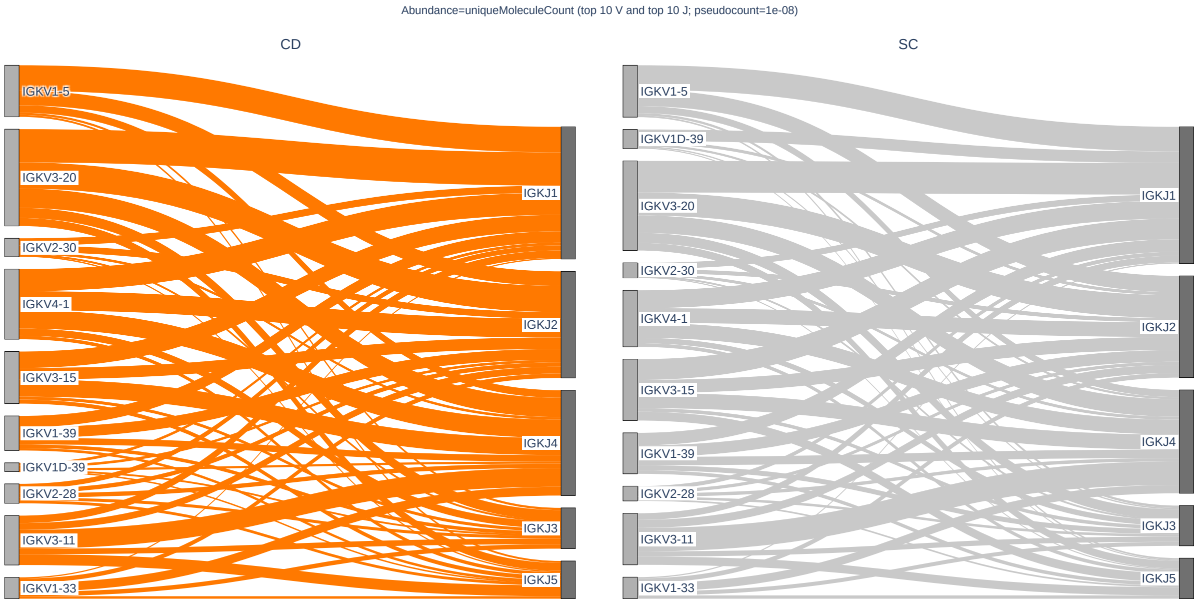

Figure S5*:* V-J gene usage in the IGK repertoire of Crohn's disease (CD) cases and symptomatic controls (SC). Sankey diagrams summarize IGK V-J recombination patterns aggregated across all productive IGK sequences in the cohort. For each diagnosis group (CD, left; SC, right), node labels represent the top 10 IGKV genes (left column of nodes) and the top 10 IGKJ genes (right column of nodes) ranked by total abundance across the entire cohort (CD+SC). Link widths are proportional to the summed abundance for each V-J pair within the indicated group (UMI counts), with a pseudo-count of 1×10^{-8}added to all V-J combinations to avoid zero values. Link colors denote diagnosis (CD, orange; SC, light grey).

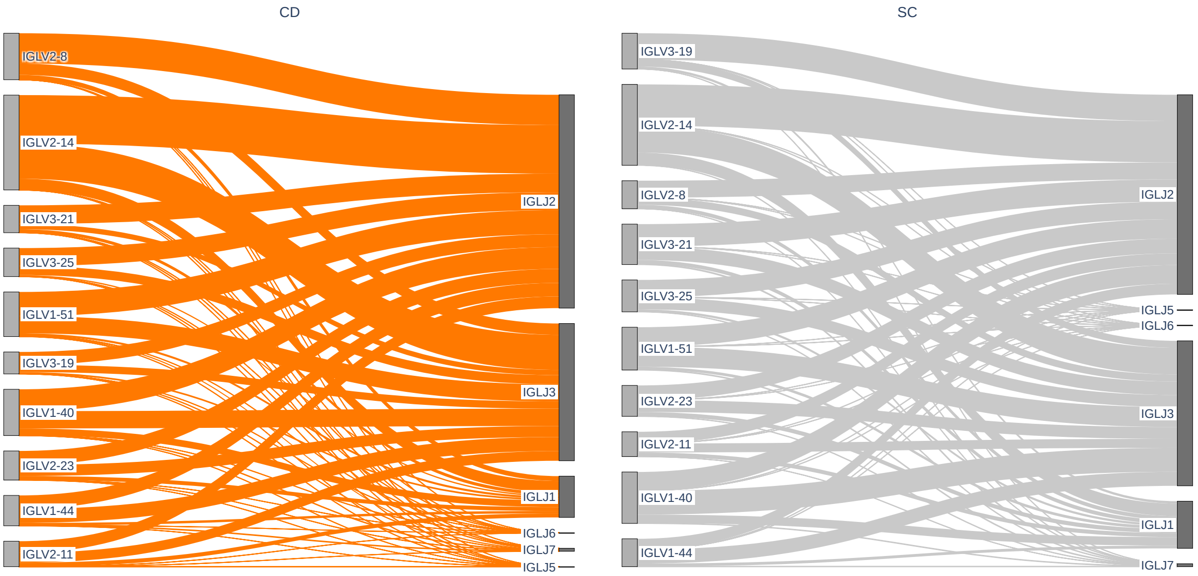

Figure S6*:* **V-J gene usage in the IGL repertoire of Crohn's disease (CD) cases and symptomatic controls (SC).** Sankey diagrams summarize IGL V-J recombination patterns aggregated across all productive IGL sequences in the cohort. For each diagnosis group (CD, left; SC, right), node labels represent the top 10 IGLV genes (left column of nodes) and the top 10 IGLJ genes (right column of nodes) ranked by total abundance across the entire cohort (CD+SC). Link widths are proportional to the summed abundance for each V-J pair within the indicated group (UMI counts), with a pseudo-count of 1×10^{-8} added to all V-J combinations to avoid zero values. Link colors denote diagnosis (CD, orange; SC, light grey).

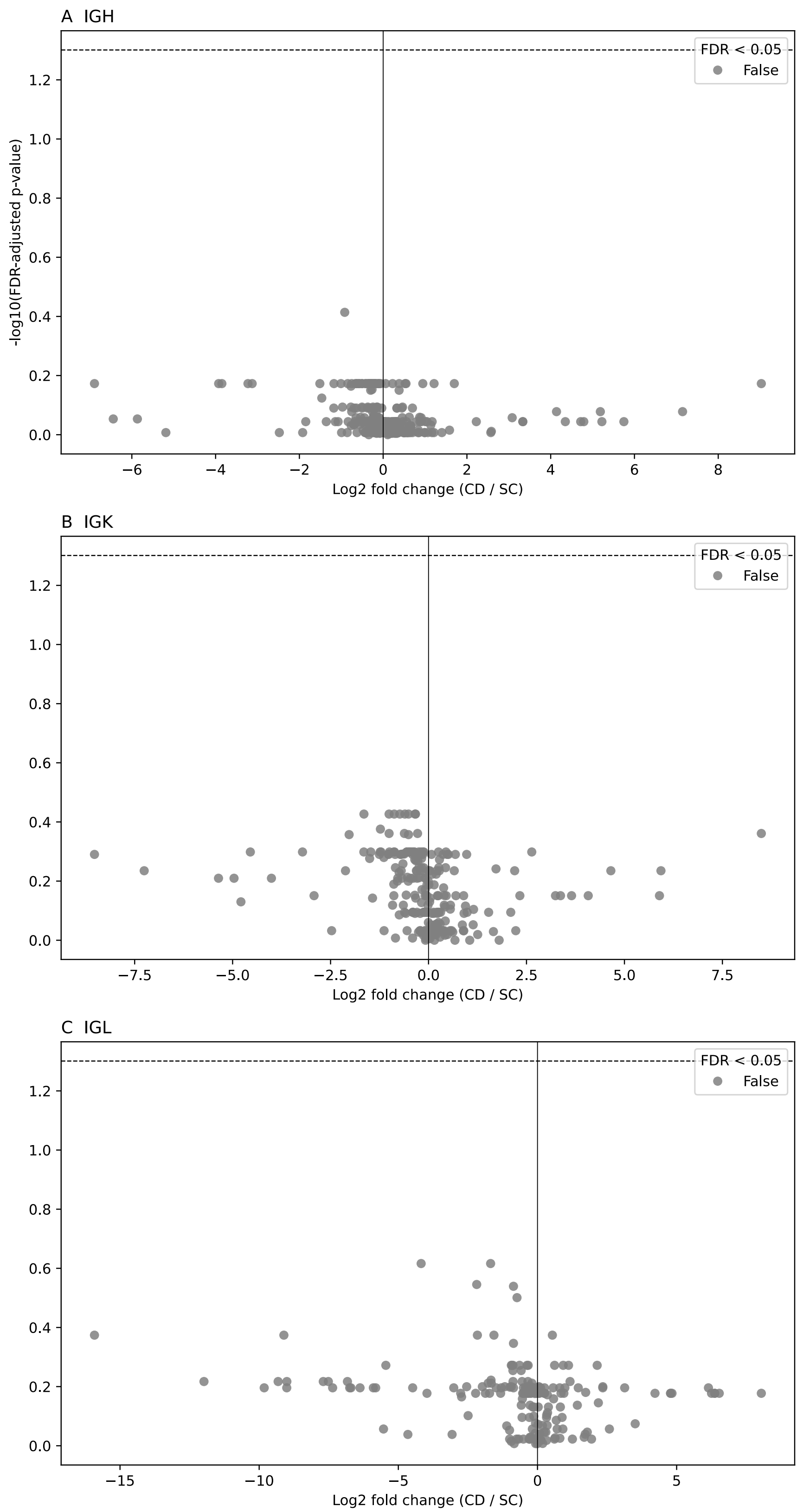

Figure S7: **Differential V-J gene usage between Crohn's disease cases and symptomatic controls across IGH, IGK and IGL**. Volcano plots summarize per-sample differences in V-J recombination usage between Crohn's disease

(CD) and symptomatic controls (SC) for the heavy chain (A, IGH), kappa light chain (B, IGK) and lambda light chain (C, IGL). For each locus, V and J calls were collapsed to gene-level (alleles removed) and V-J pairs were defined as V_gene_J_gene. Within each sample, V-J usage was quantified by summing UMI counts across all productive sequences assigned to the same V-J pair and converting to *per-sample frequencies* (VJ UMI / total UMI in that sample for the locus). For each V-J pair, CD and SC distributions were compared across samples using a two-sided Mann-Whitney U test, and p-values were adjusted for multiple testing using Benjamini-Hochberg false discovery rate (FDR). Each point represents one V-J pair; the x-axis shows the log2 fold change of mean V-J frequency (CD/SC; pseudo-count 1×10^{-8}), and the y-axis shows $-log_{10}$of the FDR-adjusted p-value. The vertical line indicates no difference (log2 fold change = 0), and the dashed horizontal line indicates the FDR significance threshold (0.05). Points are colored by significance (FDR < 0.05).
